# A Clinical Care Monitoring and Data Collection Tool (H3 Tracker) to Assess Uptake and Engagement in Mental Health Care Services in a Community-Based Pediatric Integrated Care Model: Longitudinal Cohort Study

**DOI:** 10.2196/12358

**Published:** 2019-04-23

**Authors:** Michael McCreary, Armen C Arevian, Madeline Brady, Ana E Mosqueda Chichits, Lily Zhang, Lingqi Tang, Bonnie Zima

**Affiliations:** 1 Center for Health Services and Society Semel Institute for Neuroscience and Human Behavior University of California - Los Angeles Los Angeles, CA United States; 2 Metropolitan Family Services Chicago, IL United States; 3 Erie Family Health Center Chicago, IL United States

**Keywords:** integrated health care systems, data collection methods, community-based participatory research, community mental health services

## Abstract

**Background:**

National recommendations for pediatric integrated care models include improved capacity for care coordination and communication across primary care and specialty mental health providers using technology, yet few practical, short-term solutions are available for low-resource, community-based pediatric integrated care clinics.

**Objective:**

The goal of the paper is to describe the development and features of a Web-based tool designed for program evaluation and clinician monitoring of embedded pediatric mental health care using a community-partnered approach. In addition, a longitudinal study design was used to assess the implementation of the tool in program evaluation, including clinical monitoring and data collection.

**Methods:**

Biweekly meetings of the partnered evaluation team (clinic, academic, and funding partners) were convened over the course of 12 months to specify tool features using a participatory framework, followed by usability testing and further refinement during implementation.

**Results:**

A data collection tool was developed to collect clinic population characteristics as well as collect and display patient mental health outcomes and clinical care services from 277 eligible caregiver/child participants. Despite outreach, there was little uptake of the tool by either the behavioral health team or primary care provider.

**Conclusions:**

Development of the H3 Tracker (Healthy Minds, Healthy Children, Healthy Chicago Tracker) in two community-based pediatric clinics with embedded mental health teams serving predominantly minority children is feasible and promising for on-site program evaluation data collection. Future research is needed to understand ways to improve clinic integration and examine whether promotion of primary care/mental health communication drives sustained use.

**Trial Registration:**

ClinicalTrials.gov NCT02699814; https://clinicaltrials.gov/ct2/show/NCT02699814 (Archived by WebCite at http://www.webcitation.org/772pV5rWW)

## Introduction

Pediatric primary care providers are well positioned to detect developmental delays and mental health problems early, but access to community-based mental health programs and information exchange across providers is often poor [1]. Because of this, integrating pediatric and behavioral health care has been an important part of recent efforts to enhance health care delivery and impact [2-7]. Integration facilitates increased access to behavioral health care, improved infrastructure, and enhanced financial efficiency through reorganized financial and reimbursement structures [3]. It is also associated with increased use of primary care, with patients more likely to visit their primary care doctor and make more frequent visits to primary care [8]. In integrated care, primary and mental health specialists are able to develop a relationship, helping to bring together different caregivers to develop a coordinated health care plan for patients [5]. Further, a key feature of collaborative care is tracking clinical outcomes, which has been shown to greatly increase patient response to treatment [9,10].

National recommendations for pediatric integrated care models include improved capacity for care coordination and communication across primary care and specialty mental health providers using technology [11-13]. Improved communications technology can allow therapists or other providers to be alerted when a course of treatment is ineffective or harmful, predicting treatment failure, risk of hospitalization, or other negative outcomes [9]. For patients, being able to track clinical outcomes can be encouraging, decreasing patient skepticism about the course of treatment by providing evidence of successes, thus improving patient commitment to treatment [9]. Through developing technology, three of the largest barriers to adoption of clinical tracking systems—cost to health care systems, user relevancy, and time required to learn about the tool [9]—can be mitigated because of the decreased costs associated with creating systems with this capacity, increased relevance for patients and providers through tool customization and flexibility of modification, and decreased time required to use the tool due to customization and increased user relevancy.

While the importance of coordination and communication in the health care system has been widely documented [2,4-7], these efforts are often costly, requiring system organization, privacy control, and mental and physical health system financial integration that only larger, wealthier health care organizations have [1,12,14,15]. Electronic health records (EHRs) are integral to enhancing patient care, and an effective EHR system provides more efficient contact between patients and their care team, resulting in substantial cost savings through more effective care [16-19]. When EHRs are siloed within one care system and mental and physical health services are performed through different systems, coordination of these services requires information exchange that can be infeasible for many organizations [15,19-23]. Further, the ever-changing landscape of health care can require adjustments to the EHR, and these modifications require either a large outlay of capital or internal staff with programming capabilities, less likely to be present within smaller health care organizations [23-26]. The advantages that come from EHRs, however, cannot be realized without a customized, user-centered EHR system [19,20,25,27,28].

To introduce a practical short-term solution for community-based pediatric integrated care models, this paper describes the development and implementation of the H3 Tracker, a Web-based clinical care documentation and data collection tool for use in two federally qualified health care centers with embedded mental health teams serving low-income, predominantly racial and ethnic minority children and their families. Objectives are as follows:

- To describe the development of the Web-based tool using a community-partnered participatory approach and its features;
- To assess the implementation of the tool for clinical monitoring of embedded mental health care as well as data collection for program evaluation using a longitudinal cohort study design.

## Methods

### Project Overview

Healthy Minds, Healthy Children, Healthy Chicago (H3) is a project funded by the Illinois Children’s Healthcare Foundation to improve access to and engagement in child mental health services at two federally qualified health care centers through implementation of community-based pediatric integrated care models [29]. The proposed mechanism was to integrate primary and mental health care services, improving access and engagement of these services for the prevention and early detection of children’s mental health problems [30]. Two clinic sites were selected to develop integration strategies tailored to the needs of their patient populations, staff, clinic organization, and available resources. Both clinics were located in the Chicago metropolitan area, serving low-income children and their families.

The program evaluation aims were to assess the implementation of the care models, with metrics conceptualized as uptake and engagement in mental health services, and examine the relationship between use of on-site mental health care and child- and parent-level clinical outcomes at 3, 6, and 12 months using standardized measures. To meet these aims, the H3 Tracker was developed to align with clinic workflow analyses and program evaluation design. The H3 Tracker collected data on the overall clinic population demographics; baseline and 3-, 6-, and 12-month follow-up surveys for participants; and embedded mental health or case management services provided to the children and primary caregivers enrolled in the evaluation. The study time period was 24 months, from April 2016 to March 2018. The original 1-year baseline data collection time period was extended to 18 months to improve subject enrollment. Thus, the sample of children eligible for 12-month follow-up interviews was restricted to those who completed baseline surveys within the first 12 months. Eligibility for the study was determined by a positive score on the Pediatric Symptom Checklist (PSC) corresponding to need for further mental health assessment with the option of a clinical override by clinic staff [31]. Study and informed consent procedures as well as the design and content of the H3 Tracker were approved by the institutional review boards of the University of California at Los Angeles (UCLA) and University of Illinois at Chicago. This study is also registered with ClinicalTrials.gov [NCT02699814].

### Development of the H3 Tracker

The need to develop the H3 Tracker to support program evaluation was identified during the development of the partnered evaluation process. Clinic sites and the academic team in Los Angeles explored strategies on how to transfer clinic data to the academic partner. Direct transfer from the EHR system to a secure server at UCLA was proposed; however, the resources and workforce needed to coordinate these systems and appropriately organize the data were outside of the scope of the project. Abstraction of specific data elements from existing administrative data (ie, sociodemographics) was also explored, but the team decided to have the site data coordinator (SDC) enter information directly into the H3 Tracker because overall it was less time-consuming. Further, additional elements that the evaluation required were not collected in the EHR, so creating a tool to fulfill these requirements was necessary.

Biweekly meetings were convened over the course of 12 months led by the academic partners with participation from community clinic staff and the project funder [30]. The tool was developed using a participatory framework in consultation with the principal investigator, working from a detailed evaluation design plan and clinic workflow diagrams to structure the features of the tool. Input on the development of the H3 Tracker was solicited from the data collection team, clinic physicians, mental health clinicians, and other care team members, with the majority of feedback coming from the mental health and data collection team because of their familiarity with project demands and availability. The feedback was used to refine H3 Tracker features, change wording or the ordering of questions, and add other features when possible. An iterative approach was taken, and when feature requests were unable to be incorporated due to infrastructure limitations or project needs, more feedback was solicited, with other ideas discussed and related design changes incorporated.

After sufficient discussion about project goals and H3 Tracker design, a prototype was constructed. The H3 Tracker was developed using the Chorus participatory technology platform, customized to fit the needs of a diverse clinic population as well as varying needs of the different end user types. Chorus is a Web application that allows the user to create personalized Web-based and mobile apps through a simplified visual interface without the need for computer programming skills [32,33]. The Chorus application was developed using AngularJS, Ruby, and MySQL and is a hosted service provided through UCLA. Data in Chorus are encrypted, password-protected, and stored on a secure server located at UCLA.

The prototype was presented at an in-person meeting with site data collectors and other clinic staff from both project sites, with usability tests used to inform additional modifications to the tool. During the training, participants entered mock data into the tool, and afterwards the functionality and design of each page was discussed. Particular attention was paid to aligning H3 Tracker features to the common elements of the clinics’ work flows. Together, this feedback also guided the development and refinement of the training manual.

Additionally, some areas for refinement were discovered during implementation of the tool at the clinic sites, and when possible, these were actualized during the course of the project. One was the ability to document contact attempts of participants, when the SDC would call participants to try to collect follow-up surveys. The SDCs would keep a record of their participant contact attempts on a separate document, not within the H3 Tracker, but felt that being able to document this within the H3 Tracker itself would help increase the awareness and accuracy of the documentation of previous contact attempts. Being able to respond to user demands and modify the tool accordingly helped increase the acceptability of the tool since it was more tailored to the wants and needs of users. The ability to change or add features after deployment in the clinic allowed issues to be addressed that were difficult to anticipate beforehand, which allowed for more appropriate use of the tool in the clinic workflow over what it would have been with only preimplementation user-centered design.

To assess the adoption of the tool, data on the number of user interactions were analyzed. In addition, feasibility was represented by the time spent on each page of the tool, the number of participants enrolled in the project, and the number of times a user was able to record services administered by clinic staff for each participant.

## Results

### Tool Customization: User Types and Clinics

Through discussions with academic and clinic partners, it was determined that 3 user types would be developed: (1) clinic SDC, (2) mental health clinician, and (3) pediatric primary care provider. The SDCs would input the majority of project data and record services administered to study participants. The mental health clinicians would have access to the same surveys to allow option to administer more sensitive measures (eg, trauma exposure). The primary care providers would be able to view a timeline of embedded mental health care processes for each patient as well as view the results of all of the surveys.

In addition to user types, the tool was customized for different clinic needs. Because one of the clinics served predominantly Spanish-speaking patients, a Spanish language version was created using a two-step back and forth translation approach. The tool contained validated measure translations when available, but when no validated translation existed, measures were translated by the study team.

### Tool Features and Workflow

**Figure 1:**
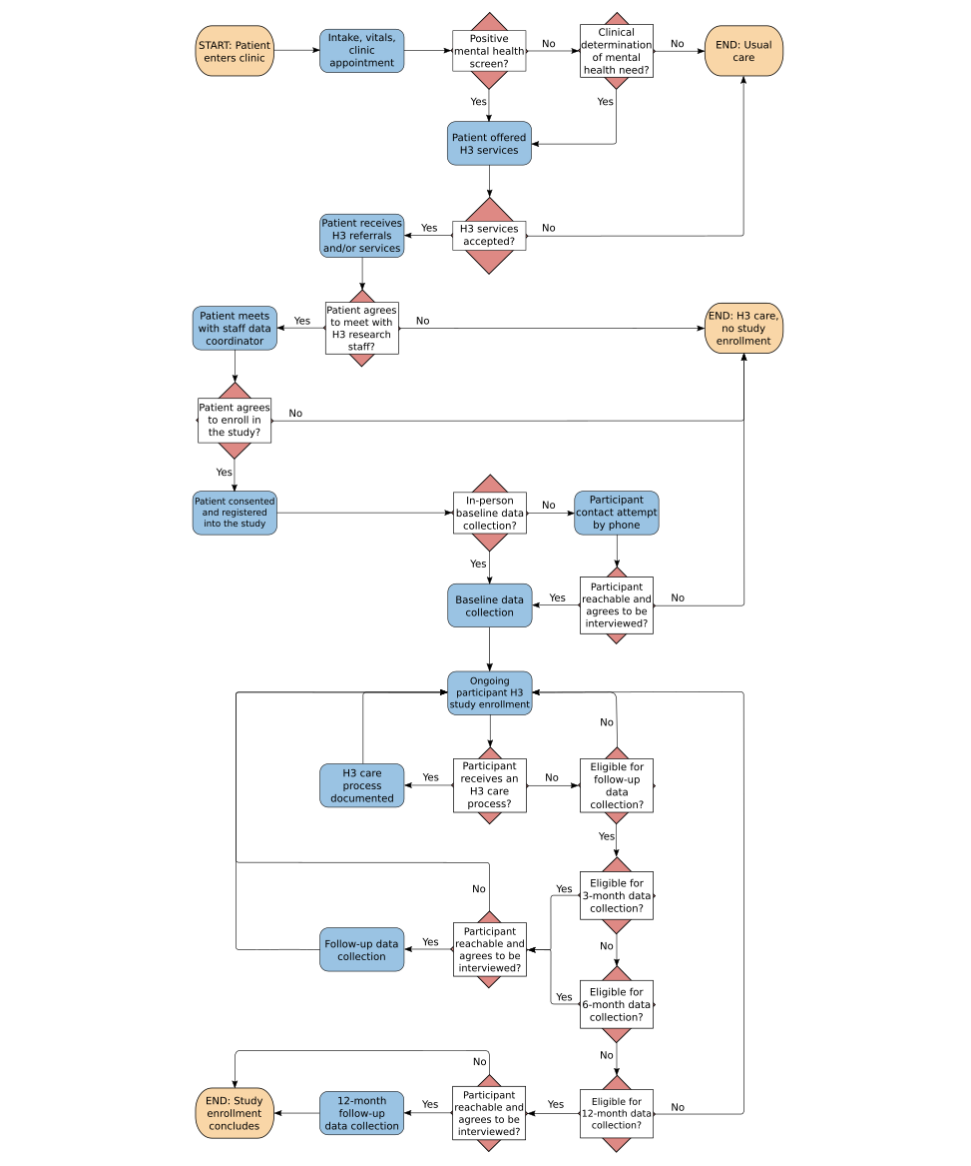
Process flow diagram. H3: Healthy Minds, Healthy Children, Healthy Chicago.

The enrollment and data collection process is summarized in Figure 1 (see Multimedia Appendices 1 and 2 for the data flow and data structure diagrams). The main features of the H3 Tracker are the Daily Census of Clinic Patients, Program Evaluation Eligibility Check, Surveys, and Clinical Care Monitoring.

#### Daily Census of Clinic Patients

The Daily Census of Clinic Patients (hereafter referred to as the Daily Census) was developed to collect general demographic information about the clinic population. In addition to demographic information, details about the child’s visit and eligibility for the study were documented. Data from the Daily Census were collected to describe the pool of eligible subjects and assess potential sources of selection bias for the evaluation.

**Figure 2:**
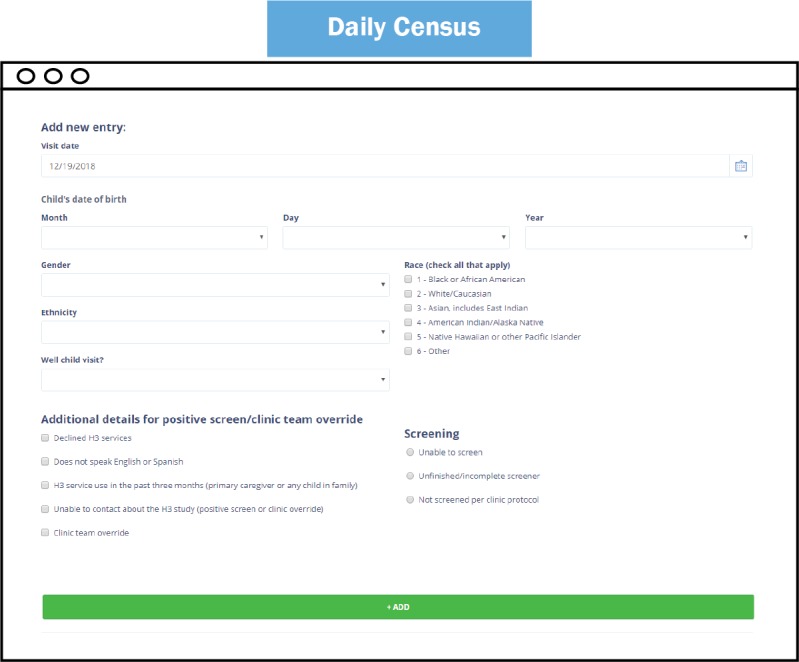
Daily Census page.

The Daily Census page (Figure 2) was modified for flexibility to the workflow of the SDCs. The assumption when the page was first developed was that the SDCs would enter patients as they came into the clinic. As project implementation progressed, it became clear that the workflow of the SDC would not often permit this; rather, the previous day’s information would be entered when the SDC did not have recruitment or other project obligations. To accommodate this change, functionality was built to allow the SDCs to edit the Daily Census date so information could be entered for patients who came in on a previous date. All of the information entered for the selected date was visible at the bottom of the screen, so that information could be tracked and, if entered incorrectly, flagged and reported. The feature to self-edit the data was not developed to limit accidental data erasures.

#### Program Evaluation Eligibility Check

The SDCs accessed the Program Evaluation Eligibility Check (Eligibility) section after a potential participant was identified. The Eligibility section was used to confirm that the patient met all eligibility criteria. Inclusion criteria were being a child aged 0 to 16.99 years and having English or Spanish language fluency and a total score on the PSC above the cut-point corresponding to need for further child mental health assessment or clinical judgement of an unmet need for child mental health care (ie, clinical override). Exclusion criteria were receipt of any on-site mental health care or case management in the past 3 months (to ensure entry of children more likely to be receiving a new episode of H3 care) and having a sibling enrolled in the study (to safeguard against clustering within families). Anyone excluded from the study could still receive H3 care at the clinic but would not be enrolled in the evaluation. If the information indicated that the patient was not eligible for the program evaluation, the entry would be flagged and a warning page would appear. Otherwise, the patient would meet with the SDC after their clinic appointment, and if there was agreement to participate, the patient would be registered in the study and baseline data would be collected. If the patient was not able to stay, the SDC would follow up with the parent by phone.

#### Surveys

After registration in the study, a reference to the parent/child dyad would appear in the Surveys section. This section contained a list of patients needing baseline or follow-up data collection, organized under the headings baseline, 3-month follow-up, 6-month follow-up, and 12-month follow-up. The lists of patients were labeled with the parent and child’s name and enrollment date, which allowed the SDC to prioritize patient outreach. Their name would appear here upon enrollment or two weeks prior to the date of the 3-, 6-, and 12-month follow-up phone interviews to allow the SDC time to contact the parent and/or youth and schedule the interview.

Upon selection of a patient in the survey list, a page appeared that allowed the SDC to log a participant contact event. If the participant was not available, the SDC would record up to 6 contact attempts until the participant was considered a soft refusal for participation, at which time the participant was removed from the list of surveys to be completed. The participant could also decline participation, and the SDC would indicate this on the page, which would remove the participant from the list.

After the SDC indicated a successful contact attempt, a list of measures would appear indicating which items needed to be collected. Data from all parent- and youth-reported measures were collected directly by the SDC with two exceptions: site 2 chose to collect data from the PSC and Traumatic Events Screening Inventory (TESI) by the on-site mental health clinicians as part of the clinic’s existing mental health screening or assessment. At both sites, these data were entered into the H3 Tracker by the SDC.

The list of measures varied by child age as well as by respondent, so the interface was programmed to only display measures relevant for the child’s age, with an indication of who should respond to the measure, the parent or the child (Table 1). The clinical outcome measures in the tool were tailored to the child’s development, addressing a unique need in pediatric integrated care models [34,35]. There were three age groups for the study, each of which received a different collection of surveys. One group was children aged 0 to 2 years. This page showed all measures except those focused on emotional and behavioral functioning of the child and trauma exposure. A second group included children aged 3 to 11 years. This page showed the emotional-, behavioral-, and trauma-focused measures for children in addition to resilience measures and measures of parental depression and parenting stress. The third group was a parent/child dyad where the child was older than 11 years. This group was given the youth version of same standardized measures for the 3- to 11-year-olds as well as a high-risk behavior measure adapted by the principal investigator to integrate clinic partners’ experience in caring for youth in their communities. After completion, the SDC would indicate that the participant had completed the baseline set of measures and surveys, which would remove the participant from the patient list of surveys to be collected.

**Table 1:** Data summary by child age group and study time point.

Measure (informant)	Child age			Time point	
	<3 yrs	≥3 yrs, <12 yrs	≥12 yrs, <17 yrs	Baseline	Follow-up
Background information (parent)	x^a^	x	x	x	–^b^
PSI-SF^c^ (parent)	x	x	x	x	x
CD-RISC-25^d^ (parent)	x	x	x	x	x
PHQ-9^e^ (parent)	x	x	x	x	x
CIS^f^ (parent)	–	x	–	x	x
PSC^g^ (parent)	–	x	–	x	x
TESI^h^ (parent)	–	x	–	x	–
Behavioral questions (parent)	x	x	–	x	x
Behavioral questions (youth)	–	–	x	x	x
Teen High Risk Behavior Survey (youth)	–	–	x	x	x
CIS (youth)	–	–	x	x	x
PSC (youth)	–	–	x	x	x
TESI (youth)	–	–	x	x	–
PHQ-9 (youth)	–	–	x	x	x
Social and behavioral health services received by patient at visit (SDC^i^)	x	x	x	x	–

^a^x: indicates measure was offered to participant subgroup.

^b^–: indicates measure was not offered to participant subgroup.

^c^PSI-SF: Parenting Stress Index Short Form.

^d^CD-RISC-25: Connor-Davidson Resilience Scale 25.

^e^PHQ-9: Patient Health Questionnaire.

^f^CIS: Columbia Impairment Scale.

^g^PSC: Pediatric Symptom Checklist.

^h^TESI: Traumatic Events Screening Inventory.

^i^SDC: site data coordinator.

#### Clinical Care Monitoring

In addition to the measures, the SDC was asked to log any mental health services or case management contacts provided during the 12-month time period of study enrollment (see Multimedia Appendix 3). Case management contacts included advocacy for special education as well as referral to and follow-up support for social services [31]. The H3 Clinical Care Monitoring (H3 Care) section allowed services to be recorded throughout a participant’s enrollment in the project, with a date assigned to each service and a timeline of services received for each patient. The services entered here were customizable, so that if a service was overlooked at the start of the project, it could be added as a new category to the list of services. This section was developed with two objectives in mind: record the use of mental health and social services by participants and provide the clinical care team a simple way to view the services received by participants in the study.

### Implementation

#### Adoption

Frequency of use of the tool was investigated to better understand how successfully it was adopted within the clinic. This helped to understand use by different user groups and determine the usefulness of various pages or features for the various users. The H3 Tracker comprised 48 pages for participants to document or track participant information with an additional 18 pages developed for provider use. Interactions with the tool were often quick, ranging from one interaction in a minute to as many as 13 interactions per minute (one interaction every 4.6 seconds).

**Table 2:** Most frequently accessed pages in the H3 Tracker.

Interaction (section)	Accessed, n (%)
Daily census (Daily Census)	21,771 (30.03)
Participant contact log (Surveys)	10,389 (14.33)
Participant list (Surveys)	9025 (12.45)
Survey list (Surveys)	7806 (10.77)
Home page (N/A^a^)	6417 (8.85)
Document clinical care service (H3 Care)	3868 (5.34)
Select patient for clinical care service documentation (H3 Care)	2398 (3.31)

^a^N/A: not applicable.

The most used feature of the H3 Tracker was the Daily Census, responsible for 30.03% (21,771/73,949) of the total interactions with the tool. Next was the participant contact documentation page in the Survey section, with 14.33% (10,389/73,949) of the total interactions on the site. Following this was the participant list page (also in the Survey section), with 12.45% (9025/73,949) of total interactions, and the survey list page, with 10.77% (7806/73,949) of total interactions. Additional pages and their uses can be observed in Table 2.

Use of the tool differed by user type, with 96.28% (71,201/73,949) of the interactions completed by the SDC, 3.71% (2744/73,949) by the integrated health assistant or family resource developer, typically filling in when the SDC was out of the office, and less than 1% (4/73,949, 0.01%) by the behavioral health counselor or social worker. For one site, there was no adoption by the behavioral health counselors, despite significant outreach, and uptake was very limited for this role at the other site. There was no adoption at either site by the primary care providers despite outreach.

#### Feasibility

The H3 Tracker was used to recruit participants, collect clinic and participant data, and collect participant service use information. A total of 20,166 visits were documented by the H3 Tracker, with 10,986 well-child visits and 9178 sick visits. There were 724 positive screens or clinical overrides, which includes 49 sick visit clinical overrides. Those who accepted H3 services numbered 507, with 340 of these recruited into the study. In total, 277 participants were enrolled in the project using the H3 Tracker and completed a baseline measure, with 1861 clinical care services documented by project staff.

Of the clients who completed a baseline survey, more than one-quarter (79/277, 28.5%) started baseline measures on the same day as their initial clinic appointment, with 24.5% (68/277) completing the baseline measures on that day, presumably using the H3 Tracker in person after their appointment. At 3-month follow-up, 66.8% (185/277) completed a phone interview, consistent with other similar studies [36-38]. In addition, the 6- and 12-month follow-up response rates were 59.2% (164/277) and 53.3% (56/105) respectively.

### Sample Characteristics

The majority of children in the study were from racial and ethnic minority backgrounds (site 1: Latino 192/202, 95.1%; site 2: African American 71/75, 95%), consistent with general clinic population characteristics. Children in the study were predominantly English speaking, with 75.8% (210/277) reporting their primary language as English. Caregivers were mostly female (263/277, 94.9%) while the gender of children was more evenly distributed, with slightly more male than female (male: 152/277, 54.9%; female: 125/277, 45.1%). Caregivers were most often the parent of the child, with 95.6% (263/275) of cases reported to be the mother or father, 3.6% (10/275) grandparent, and less than 1% (1/275, 0.4%) other legal guardian (nonrelative). Most of the children were younger than 12 years (214/277, 77.3%) and 32.9% (91/277) were younger than 6 years, with a mean age of 8.1 (SD 4.2) years.

## Discussion

### Principal Findings

Findings from this study suggest that it is feasible to develop a Web-based clinical monitoring and data collection tool using a community-partnered participatory approach for two pediatric integrated care models serving low-income, predominantly racial and ethnic minority children and their families [33]. Through an iterative, partnered approach, capacity was built within the H3 Tracker to serve these dual purposes. Using the Chorus platform enabled the app to be developed visually by study staff, eliminating the need for involvement of computer programmers in the development process. The H3 Tracker had the built-in capacity to allow the primary care/mental health team to chronologically track the delivery of on-site mental health services and case management activities as well as clinical outcomes using standardized child, youth, and parent-reported measures, consistent with national recommendations for a pediatric medical home [39]. Further, uptake and use of the main features for data collection at baseline and 3 follow-up time points during pilot testing of the care models was highly promising.

Additional modifications could increase adoption, use, and capacity of the H3 Tracker. Having the data integrated into the existing EHR systems could facilitate easier access to the information, increasing the perceived utility of the EHR [21,40,41] as well as its adoption and use [42]. While input from clinicians was obtained during the development process, a more engaged approach to obtaining clinician feedback on the tool may be appropriate. Further, including a liaison in integrated care systems has been identified as a key factor to the development and operationalization of integrated care [43], leading to more effective care integration [44]. Therefore, another step would be to examine whether a technology liaison may improve clinician engagement with the tool to help integrate quality-of-care monitoring into clinical practice. Other modifications of the H3 Tracker could include expanding the main features to accommodate capturing the workflow of usual care processes in addition to mental health and social services and building capacity to communicate health information directly to patients. Future research is needed to develop approaches that facilitate clinician use, reduce end-user burden, and better align with existing workflow, EHR documentation, and billing requirements.

### Limitations

Findings from this study should be interpreted in light of several limitations. The purpose of the larger study was to evaluate the care model, so we did not collect the number of screened participants. Further, although capacity was built for three end users, adoption of the H3 Tracker varied by purpose and user groups. Uptake and use as a data collection tool far exceeded use of the tool’s capacity to monitor mental health care delivered and clinical outcomes. Low uptake by clinical staff hindered the fidelity of the study, as large parts of the tool went mostly unused. The tool was designed for a research project and not as a permanent feature of clinic workflow, and time demands may have made it difficult for the clinical staff to learn a new tool, given that it would only be a temporary part of clinic processes. In addition, the tool tracked information on a small fraction of the total clinic population, as most of the information collected by the H3 Tracker was only for those enrolled in the study. A number of studies have outlined the difficulties faced by busy clinic staff in incorporating new technology into their workflow, with usefulness as a key indicator for adoption of the technology [15,24,42].

### Conclusions

Overall, a tool like the one described here could help assist health care organizations in need of collaborative care coordination but lacking the institutional capacity of larger health care systems. The tool provides a secure, low-cost solution that staff with little to no computer programming experience can develop, tailored to the specific needs of each user and individual clinic, hospital, or other health care facility. The simplicity of the development process could allow multiple care team members to help develop features of the system, which can lead to improved customization to the needs of individual clinics and/or the type of user accessing the system. This leads to a system that is more representative of users’ needs, leading to improved usability.

The goal of building a comprehensive system where physical and mental health care is seamlessly integrated is still a very difficult task for many health care providers. Systems such as the one discussed here could be an important bridge to promoting communication across pediatric primary care providers and an embedded mental health team while the hurdles associated with full-scale integration, cost issues, system specifications, type of system to use, and data security and storage are resolved.

## Appendix

Multimedia Appendix 1 Data flow diagram.

Multimedia Appendix 2 Data structure diagram.

Multimedia Appendix 3 Clinical care monitoring services recorded by the H3 Tracker.

## Abbreviations

EHR: electronic health record
H3: Healthy Minds, Healthy Children, Healthy Chicago
PSC: Pediatric Symptom Checklist
SDC: site data coordinator
TESI: Traumatic Events Screening Inventory
