# Supplementary figures and images for: A Clinical Care Monitoring and Data Collection Tool (H3 Tracker) to Assess Uptake and Engagement in Mental Health Care Services in a Community-Based Pediatric Integrated Care Model: Longitudinal Cohort Study
Source: JMIR Ment Health. 2019 Apr 23;6(4):e12358. doi: 10.2196/12358 (PMC6658269; doi:10.2196/12358)

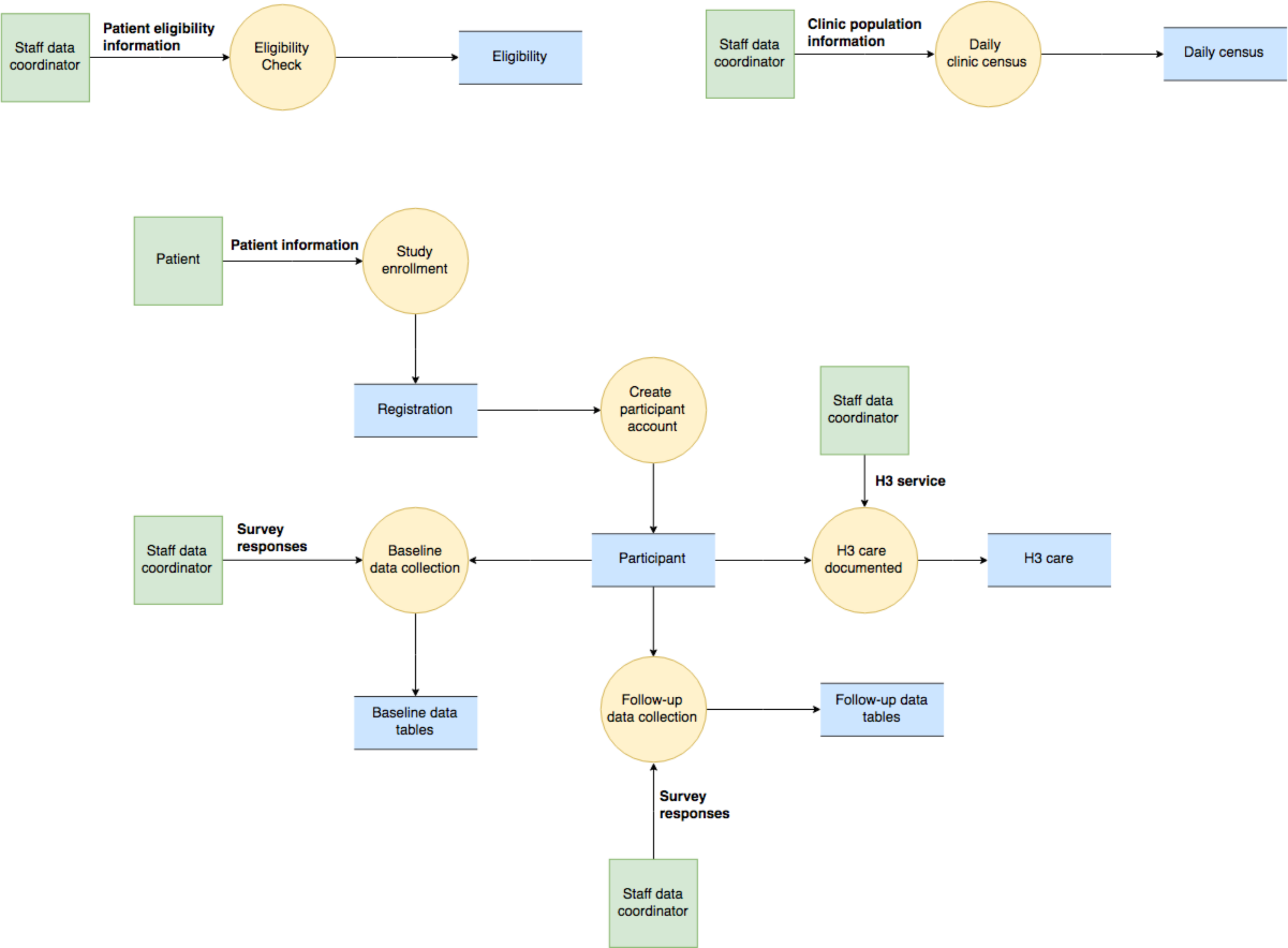

Supplement: Multimedia Appendix 1 [file mental_v6i4e12358_app1.pdf]
