# Supplementary material for: A Clinical Care Monitoring and Data Collection Tool (H3 Tracker) to Assess Uptake and Engagement in Mental Health Care Services in a Community-Based Pediatric Integrated Care Model: Longitudinal Cohort Study
Source: JMIR Ment Health. 2019 Apr 23;6(4):e12358. doi: 10.2196/12358 (PMC6658269; doi:10.2196/12358)
Supplement: Multimedia Appendix 2 [file mental_v6i4e12358_app2.pdf]

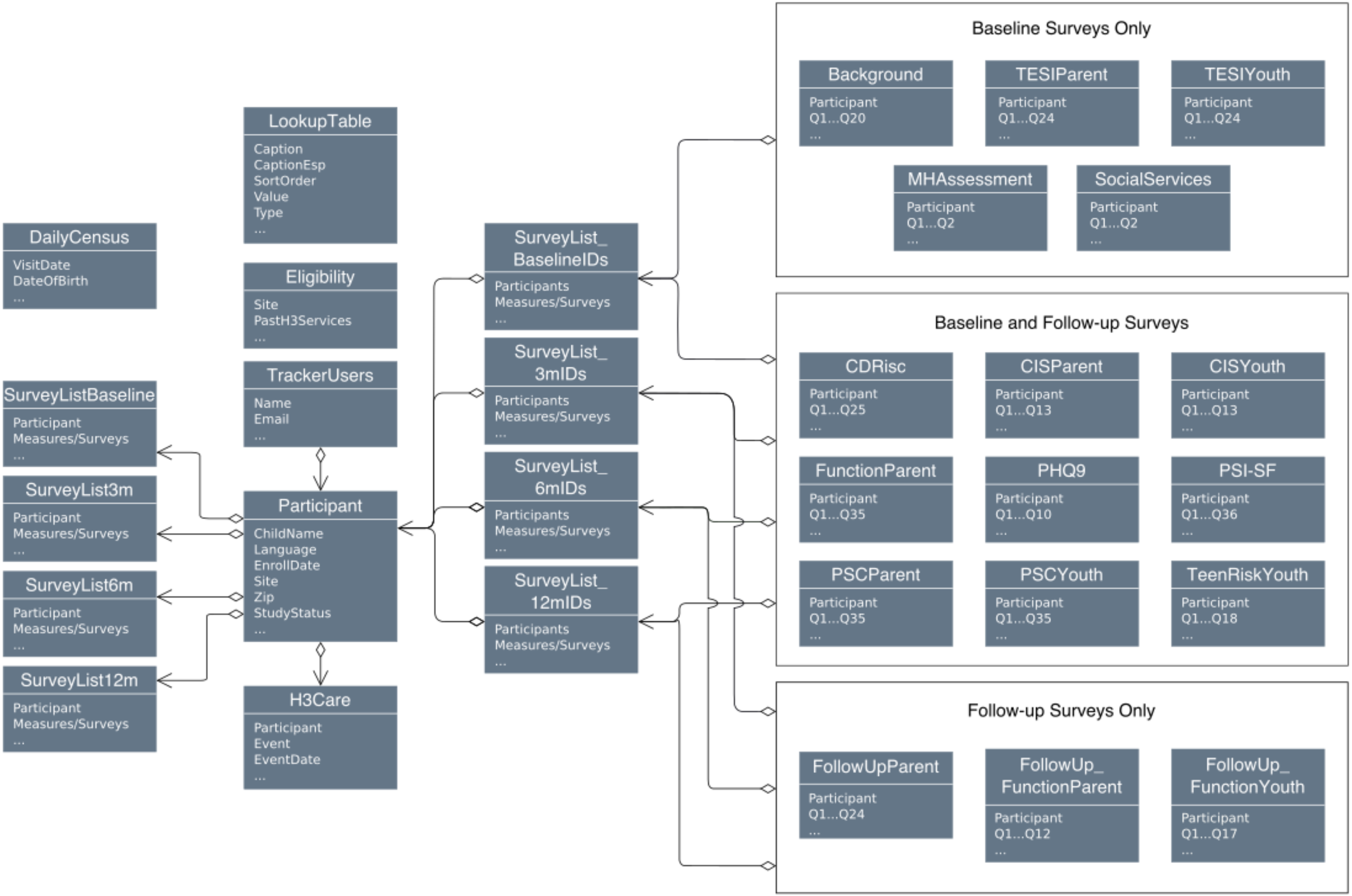

Note: Elements of this diagram have been simplified for ease of viewing and space considerations. The "LookupTable" above connects to all other tables to allow each field to have multiple characteristics. For example, when displaying the text for a measure to be collected, the answer choices can be in either English or Spanish but will reference identical fields in the database regardless of the text that was displayed. In addition, the fields within each variable have all been truncated, and the question fields (i.e. Q1...Q24) within each survey variable have been abbreviated with ellipses to denote the total number of questions for each survey variable without listing each field separately.
