# Supplementary material for: A Clinical Care Monitoring and Data Collection Tool (H3 Tracker) to Assess Uptake and Engagement in Mental Health Care Services in a Community-Based Pediatric Integrated Care Model: Longitudinal Cohort Study
Source: JMIR Ment Health. 2019 Apr 23;6(4):e12358. doi: 10.2196/12358 (PMC6658269; doi:10.2196/12358)
Supplement: Multimedia Appendix 3 [file mental_v6i4e12358_app3.pdf]

Crisis Intervention – Instigation of crisis intervention protocols at the clinic, such as referral for assessment for inpatient psychiatric care, immediate intervention to address concerns regarding safety, or grave disability due to mental illness

Medication Evaluation or Treatment – PCP or CAP – Any treatment provided by a primary care provider or Child and Adolescent Psychiatrist that involves evaluating the child for a potential need for psychiatric medication

Parent Training – Attending a course of Parenting Fundamentals or the EZ Parent program, two parenting classes hosted by the clinics

Parenting Support – Emotional support, empathy provided to parent

Psychoeducation – Includes parent education about development, approaches to a child if exposed to trauma, preventive interventions

Short-term Therapy – Trauma focused CBT or brief psychotherapies using Practice Wise (CBT for anxiety, CBT for depression, parent management for Conduct and ADHD, sleep hygiene)

Social Services – Case Management – Any contact with the Behavioral Health Coordinator or Integrated Health Assistant to follow up on referrals or services

Special Education Advocacy and Support – Any recommendations, advice or information provided to a family related to help with Special Education programs or services

Specialty Mental Health Visit – Onsite – Any onsite visit to specialty mental health care for the child or family that occurs

Referral to DD services – Referral to developmental delay programs and general hospitals for developmental delay services

Referral – Medication Evaluation – Psychiatrist – Referral to a psychiatrist for the purpose of receiving a medication evaluation for the child

Referral – Specialty Mental Health for Parent – Parent or caregiver referral to a specialty mental health provider

Referral – Parent Training – Referral to a parent training program

Referral – Social Services – Economic Support – Referral for services to help with housing, employment, food, clothing

Referral – Social Services – Early Education – Information on education related supports.

Referral – Social Services – Legal Aid – Any referral for legal services or legal help

Referral – Social Services – Other – Any other social service related referral not covered by Legal Aid, Early Education, or Economic Support

Referral – Specialty Mental Health – Warm Handoff, Co-located – Referral to a specialty mental health provider, often triggered after a trial of short-term therapy. Co-located refers to services within the same building as the clinic

Referral – Specialty Mental Health – Warm Handoff, Affiliated Site – Referral to a specialty mental health provider, often triggered after a trial of short-term therapy. Affiliated site is any site within the clinic or within partnering organizations

Referral – Specialty Mental Health – Warm Handoff, Non-affiliated – Referral to a specialty mental health provider, often triggered after a trial of short-term therapy. Non-affiliated is any non-affiliated site
